# The Mental Deficiency Act, 1913: The General Scope of Its Provisions

**Published:** 1913-10-11

**Authors:** G. E. Shuttleworth


### THE MENTAL DEFICIENCY ACT, 1913.

### The General Scope of its Provisions.

BY G. E. SHUTTLEWORTH, M.D., M.R.C.S.

On August 15 the Royal Assent was given to the Mental Deficiency Act, an Act which, for the first time, extends State protection and control in England and Wales, as does a corresponding Act for Scotland, to a class of the population not hitherto amenable to the Lunacy Laws, though to a large extent incapable, through congenital mental defect, of taking a responsible position in the social economy of the nation. For several decades past social workers have been impressed with the frequent occurrence of these feckless, anomalous beings whom philanthropic efforts have failed to raise from a dependent, frequently delinquent, position; and the full investigation made by the Royal Commission on the Care and Control of the Feeble-Minded, lasting from 1904 to 1908, established the fact of the existence in our midst of "numbers of mentally defective persons whose training is neglected, over whom no sufficient control is exercised, and whose wayward and irresponsible lives are productive of crime and misery, of much injury and mischief to themselves and to others, and of much continuous expenditure wasteful to the community and to individual families." Remedies for this state of things were suggested by the Commission, but owing to the exigencies of party strife opportunity was not found for Parliamentary action until 1912, when Government introduced a Bill dealing with the subject. A single session did not suffice for its discussion; but, reintroduced in an amended form the following session, the Act will come into full operation on April 1, 1914.

The Act contains 72 sections, and the résumé following gives a brief account of its main provisions:—

### I. Power and Manner of Dealing with Defectives.

Defectives within the meaning of the Act are defined under four headings, viz.:—

(*a*) Idiots; that is to say, persons so deeply defective in mind from birth or from an early age as to be unable to guard themselves against common physical dangers;

(*b*) Imbeciles; that is to say, persons in whose case there exists from birth or from an early age mental defectiveness not amounting to idiocy, yet so pronounced that they are incapable of managing themselves or their affairs, or, in the case of children, of being taught to do so;

(*c*) Feeble-minded persons; that is to say, persons in whose case there exists from birth or from an early age mental defectiveness not amounting to imbecility, yet so pronounced that they require care, supervision, and control for their own protection or for the protection of others; or, in the case of children, that they by reason of such defectiveness appear to be permanently incapable of receiving proper benefit from the instruction in ordinary schools.

(*d*) Moral imbeciles; that is to say, persons who from early age display some permanent mental defect coupled with strong vicious or criminal propensities on which punishment has had little or no deterrent effect.

Powers are given to parents or guardians to send to institutions for defectives, or to place under guardianship, idiots and imbeciles, and to parents (only) similarly to place their feeble-minded offspring if under the age of twenty-one. Public authorities may also deal with those who, in addition to being defectives, are persons "neglected, abandoned, or without visible means of support, or cruelly treated"; or found guilty of any criminal offence, or liable to be sent to an industrial school; or undergoing imprisonment (other than under civil process) or penal servitude or detention in reformatories, industrial schools, inebriate reformatories or mental hospitals; or habitual drunkards under the Inebriate Acts; or reported by local education authorities as incapable of improvement or requiring detention after probation in special schools; or mothers of illegitimate children receiving poor relief.

To the local education authority, subject to regulations made by the Board of Education, is assigned the duty of notifying the local authority in the case of all defective children in their area, over the age of seven, who require permanent care and control. Two medical certificates, one of which is to be signed by "a medical practitioner approved for the purpose by the local authority or the Board," are required in the case of idiots and imbeciles placed in institutions or under guardianship by their parents or guardians, and in the case of a feeble-minded person under twenty-one so placed by his parent the signature of a judicial authority is also required. Thus great care has been taken to safeguard the liberty of the subject. In cases where defectives are dealt with otherwise than at the instance of parents or guardians it is by orders made by judicial authorities, courts of law, or the Home Secretary.

### II. Central and Local Authorities.

For the general superintendence of matters relating to the supervision, protection, and control of defectives a central authority is set up, designated "the Board of Control," and this is to consist of not more than fifteen commissioners, not more than twelve to be paid, and, of the latter, four are to be legal and four medical commissioners, and "at least one of the paid and one of the unpaid commissioners shall be a woman." The legal commissioners only are to be appointed by the Lord Chancellor; the rest by the Home Secretary, who will also appoint the paid chairman. In the first instance the present eight paid Commissioners in Lunacy will become paid members of the Board of Control, so that there will be four paid vacancies to fill. No conditions of qualification are laid down for these new appointments; but to enable the holders to take part in duties under the Lunacy Acts similar qualifications to those required of the present paid Lunacy Commissioners would seem to be necessary, as it must be remembered that those Acts, as well as the new Act, will be administered by the Board of Control. The appointment of subordinate inspectors is also provided for.

As regards local authorities, which will consist of the County or County Borough Councils, the duty is imposed on them of constituting in each district a "Special Committee for the Care of Defectives," of which some members must be women. Their general powers and duties will be to ascertain, except in the case of those dealt with at the instance of their parents or guardians (under Section 2, 1 (a)), what persons within their area are defectives subject to be dealt with under the Act; to provide suitable supervision for such persons and, if necessary, to place them in institutions or under guardianship; to maintain them, wholly or in part, in such institutions or under guardianship; and to make such reports as the Board of Control may require. Reservations are, however, made (i) contingent on the Treasury grants being less than one-half of the total expenses incurred; (ii) in the case of those dealt with by Poor-Law authorities and (iii) under the Lunacy Acts, and (iv) in the case of children between seven and sixteen years of age, the functions of the local education authority are not to be interfered with.

III. Provision of Institutions and Certification.

The institutions contemplated in the Act fall under four categories, viz.:—

1. State institutions (for defectives of dangerous or violent propensities) to be established and managed by the Board of Control at the public expense.
2. Certified institutions or houses, charitably or privately founded and maintained, to be certified and inspected by the Board, and to possess powers of detention under conditions laid down in the Act.
3. Poor-Law institutions under Guardians, to be approved and certified by the Board.
4. Approved homes (without power of detention) to be approved by the Board subject to such conditions as they may think fit.

It is to be noted that Section 51 makes it a misdemeanour to undertake the care and control of more than one defective elsewhere than in a certified institution or house or an approved home.

Space will not permit further details of the Act, but our readers will have before them the general scope of its provisions, which, if wisely administered, cannot fail to benefit the afflicted (and too frequently misunderstood and neglected) class for whom it is intended. It must be remembered that the persons with whom it will deal are the subjects of congenital defect, and that much of the wild talk that has been current as to the Act rendering it possible to immure for life mere "innocents" is devoid of foundation. The majority of those to be dealt with under the Act will probably be those already brought into contact with existing law, if we include under that term children ascertained to be defective under the special Education Act of 1899, which still requires to be made universally compulsory.

Special schools afford the best available means of discriminating between the improvable and the unimprovable class of defectives; indeed, in many young cases a period of probation therein is essential to ascertain whether apparent defect will be permanent or not. To speak of industrial colonies for defectives as "prisons" is simply an abuse of language, as may be seen by a perusal of the reports of, or preferably a visit to, that established by the Lancashire and Cheshire Society at Sandlebridge.* Here nearly 300 boys and girls (many from the slums of Manchester) enjoy healthy, happy lives in rural surroundings, the younger ones being trained in such scholastic studies as they are capable of, and those over sixteen working in the gardens, on the farm, and in the laundry. It is satisfactory to note that the last published report states that the farm and gardens yielded a profit of £538 in 1912 after paying a rent of £180 to the Society.

* Miss Dendy, Hon. Secretary of this Society, contributes an article on its work on page 37.

